# Viral Coinfection and Nasal Cytokines in Children With Clinically Diagnosed Acute Sinusitis

**DOI:** 10.3389/fped.2021.783665

**Published:** 2022-01-12

**Authors:** Santiago M. C. Lopez, Nader Shaikh, Monika Johnson, Hui Liu, Judith M. Martin, John V. Williams

**Affiliations:** ^1^Department of Pediatrics, Sanford School of Medicine, University of South Dakota, Sioux Falls, SD, United States; ^2^Sanford Research, Environmental Influences on Health and Disease Group, Sioux Falls, SD, United States; ^3^Department of Pediatrics, University of Pittsburgh School of Medicine, University of Pittsburgh Medical Center Children's Hospital of Pittsburgh, Pittsburgh, PA, United States

**Keywords:** sinusitis, virus, mucosal immune response, cytokines, children

## Abstract

**Objective:** Children with no pathogenic bacteria in the nasopharynx are unlikely to have acute bacterial sinusitis. We evaluated whether information on clinical presentation, viral co-detection, and mucosal cytokine levels could be used to predict presence of bacteria in the nasopharynx.

**Method:** We obtained nasopharyngeal (NP) swabs from children diagnosed with acute sinusitis. NP swabs were processed for bacterial culture, viral PCR testing, and cytokine expression. We examined whether results of the bacterial culture could be predicted based on the presence of clinical information, presence of viruses or mucosal cytokine levels.

**Results:** We enrolled 174 children; 123 (71%) had a positive culture for potentially pathogenic bacteria and 51 (29%) had normal flora. 122/174 (70%) tested positive for one or more viruses. Compared to children with normal flora, children with pathogenic bacteria were more likely to have viruses (*p* < 0.01), but this relationship disappeared when we adjusted for age. Children with pathogenic bacteria in their nasopharynx and children with normal flora had similar levels of nasal cytokines.

**Conclusion:** In children with clinically diagnosed acute sinusitis, clinical presentation, levels of nasal cytokines, and presence of viruses do not differentiate children with and without pathogenic bacteria in their nasopharynx.

## Introduction

Acute sinusitis is one of the most common infections in childhood leading to antibiotic prescription (1, 2). Differentiation between acute sinusitis requiring antibiotics and an uncomplicated viral upper respiratory tract infection is currently based on the duration of upper respiratory tract symptoms. Thus, the tools to diagnose and manage this condition remain limited. Accordingly, if ancillary tests could differentiate children with bacterial acute sinusitis and children with an uncomplicated viral infection, unnecessary use of antimicrobials could be reduced. This is especially important since acute sinusitis is a leading cause of outpatient antimicrobial prescription (1–3).

Previous studies have shown that, for children with acute otitis media, which has very similar pathogens compared to acute sinusitis, when pathogens are absent from the nasopharynx, bacterial superinfection is highly unlikely (4–8). In this study, we aimed to investigate whether nasal cytokine levels, clinical presentation, or presence of viruses, which could in principle be performed as a point of care test, could predict nasopharyngeal bacterial culture, which takes several days to result.

## Methods

### Participants

Subjects were children 2–12 years of age who were participants in an ongoing multicenter prospective clinical trial (www.ClinicalTrials.gov, study identifier: NCT02554383). We used stringent diagnostic criteria, defined *a priori*, consistent with the American Academy of Pediatrics guidelines (9). Eligible subjects had either: (1) persistent upper respiratory tract infection symptoms, i.e., 10–29 days of cough (not exclusively nocturnal) and/or nasal symptoms (rhinorrhea of any quality) which was not improving; or (2) worsening symptoms (substantial worsening of nasal symptoms and/or fever after a period of improvement). We excluded children who had: (1) severe presentation (9); (2) had received antimicrobial treatment within 15 days before presentation; (3) had evidence of another infection (i.e., acute otitis media or pneumonia); or (4) had underlying immune deficiency, cystic fibrosis, ciliary dyskinesia, or major developmental delay. The University of Pittsburgh Institutional Review Board approved the study and parents/guardians provided written consent.

At the time of diagnosis, we collected detailed information about the child's presenting symptoms (number of days with symptoms, fever, nasal discharge, daytime cough, asthma, seasonal allergies, local and systemic antihistamine use, exposure in school, and season of diagnosis). In addition, parents answered a previously published validated electronic measure of symptom severity [Pediatric Rhinosinusitis Symptoms Scale (PRSS)] (10).

## Materials: Samples Collection, Microbiology, and Cytokines Data

### Specimen Collection and Processing

We obtained a NP sample from one nostril using a sterile, flexible, thin, flocked swab (ESwab™ -comprises 1 mL of Liquid Amies medium and a FLOQswab™-, COPAN Diagnostics Inc.). The tip of the nose was raised, and the swab introduced gently along the floor of the nasal cavity, passing under the inferior turbinate until the pharyngeal wall was reached. Once the swab was in contact with the pharyngeal wall it was removed gently. The swab was processed and refrigerated at 2–8°C until transport to the laboratory on ice. On arrival to the microbiology laboratory, it was processed into several aliquots (11) for viral detection, semi-quantitative bacterial culture, and cytokine transcript levels.

### Bacterial Identification

For bacterial culture, samples were plated on three types of media: trypticase soy agar, 5% sheep blood agar, and chocolate agar (Becton Dickinson). Cultures were incubated overnight at 37°C with 5% CO_2_. If no growth was present after overnight incubation, the culture was reincubated for another 24 h. Growth of pathogenic bacteria (*Streptococcus pneumoniae, non-typeable Haemophilus influenzae, Moraxella catarrhalis*) was assessed using standard techniques (12).

### Viral Identification

We performed individual real-time RT-PCR assays for the detection and subtyping of human rhinovirus (HRV), influenza subtypes A/B/C, adenovirus, human metapneumovirus (HMPV), parainfluenza virus (PIV) subtypes 1–4, and respiratory syncytial virus (RSV) using previously described methods (13–15). Negative and positive controls were included with each run and specimens were considered positive if the CT value was <40 cycles.

### Cytokine Measurement

Nucleic acid extraction was performed with the ABI MagMax96 Express automated instrument. Gene expression of interleukin (IL)-6, IL-8, IL-10, IL-13, IL-17, IL-22, IL-25, IL-26, sIL22RA2, IL-33, interferon (IFN) α, β, γ, and λ was measured using exon-spanning primers and probes according to the manufacturer's instructions (TaqMan™, Applied Biosystems). All values were normalized to the glyceraldehyde 3-phosphate dehydrogenase (GAPDH) housekeeping gene. Cytokines were grouped into three functional classifications based on published data: proinflammatory (IL-6, IL-8, IL-17, IL-22, IL-25, IL26, and IL-33), anti-inflammatory (IL-10, IL-13, and sIL22RA2) and antiviral (IFN-α, IFN-β, IFN-γ, and IFN-λ) (16–25). We normalized cytokine expression using the 2^−ΔΔCT^ method (26), by enrolling 30 age-matched asymptomatic healthy controls who had no underlying medical conditions. We excluded children with fever or cold symptoms within the past 7 days; antimicrobial use in the last 14 days; or nasally administered medication in the last 15 days. There was no difference on the mean age, gender and viral detection of asymptomatic healthy controls compared with the study group. Further data regarding this group are not shown.

## Data Analysis

### Statistical Analysis

We used Kruskal-Wallis test for continuous variables and Chi-squared or Fisher's exact tests for categorical variables. Because 14 cytokines were tested, we used Benjamini-Hochberg correction to adjust *p*-values for multiple comparisons. For the multivariate analysis, we performed a logistic regression model with forward selection. Two-sided *p* < 0.05 were considered statistically significant throughout. All statistical analyses were performed using SAS 9.4 and R 3.6.2.

## Results

### Subject Characteristics

We enrolled 174 children with clinically diagnosed AS. The median age of the children was 4.9 years [IQR: 3.5, 7.5]. A total of 123/174 (71%) had a positive bacterial culture for potentially pathogenic bacteria (i.e., *Streptococcus pneumoniae, non-typeable Haemophilus influenzae, Moraxella catarrhalis*). These children will hereinafter be referred to as being “colonized with bacteria.” A total of 51 (29%) children had growth of normal respiratory flora (hereinafter referred children with “normal flora”).

**Figure 1:**
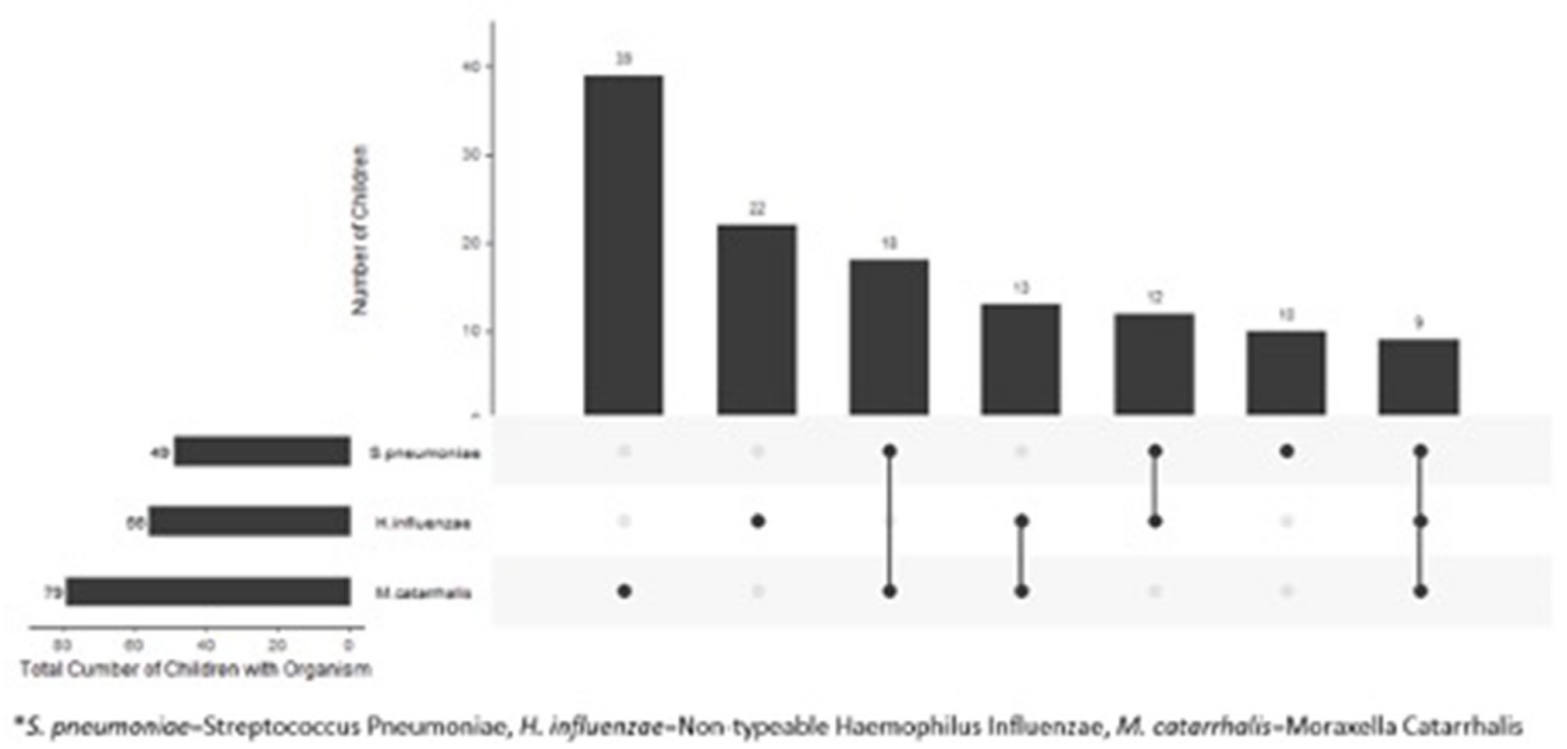
Distribution of bacteria in 123 children with clinical diagnosis of acute sinusitis who had pathogens in their nasopharynx.

Compared to children with normal flora, children colonized with bacteria were younger (4.2 vs. 7.6 years, *p* < *0.01*) and were more likely to be non-white (*p* = *0.03*) (Table 1). We found no difference in gender or clinical presentation between two groups (Table 1). Among children colonized with bacteria, 71/123 (58%) were colonized with a single pathogen and 52 (42%) were colonized with >1 pathogen. The most frequently cultured bacteria was *Moraxella catarrhalis* (*n* = 79) (Figure 1).

**Table 1:** Demographics and clinical characteristics of children with acute sinusitis.

	**Colonized with bacteria**	**Normal flora**	***P*-value**
	**(*n* = 123)**	**(*n* = 51)**	
**Age, years**			
Median [IQR]	4.2 [3.2, 6.0]	7.6 [4.9, 9.5]	<0.01
**Gender**			
Male	65 (53%)	24 (47%)	0.49
Female	58 (47%)	27 (53%)	
**Race**			
White	59 (48%)	36 (71%)	0.03
Black	47 (38%)	11 (22%)	
Other	17 (14%)	4 (8%)	
Number of days with symptoms median [IQR]	14.0 [10.0, 15.0]	14.0 [11.0, 14.0]	0.74
**Fever/felt warm**			
Yes	61 (50%)	27 (53%)	0.69
No	62 (50%)	24 (47%)	
**Green or yellow nasal discharge**			
Yes	85 (69%)	32 (63%)	0.42
No	38 (31%)	19 (37%)	
**Daytime cough**			
Yes	120 (98%)	49 (96%)	0.63
No	3 (2%)	2 (4%)	
**Asthma**			
Yes	27 (22%)	10 (20%)	0.73
No	96 (78%)	41 (80%)	
**Seasonal allergies**			
Yes	40 (33%)	18 (35%)	0.72
No	83 (67%)	33 (65%)	
In school/preschool^*^			
Yes	99 (80%)	40 (78%)	0.76
No	24 (20%)	11 (22%)	
**Season of diagnosis**			
Spring	48 (39%)	26 (51%)	0.48
Summer	8 (7%)	4 (8%)	
Fall	34 (28%)	11 (22%)	
Winter	33 (27%)	10 (20%)	
**PRSS**			
Median [IQR]	24.0 [19.0, 30.0]	25.0 [18.0, 29.0]	0.86
**On oral antihistamine^‡^**			
Yes	19 (15%)	9 (18%)	0.72
No	104 (85%)	42 (82%)	
**On nasal antihistamine^‡^**			
Yes	2 (2%)	1 (2%)	>0.99
No	121 (98%)	50 (98%)	

*IQR, Interquartile range; PRSS, Pediatric Rhinosinusitis Symptom Score*.

* *With at least ≥6 children for ≥10 h per week*.

‡ *Within 1 week of enrollment*.

### Viral Detection During Acute Sinusitis

**Figure 2:**
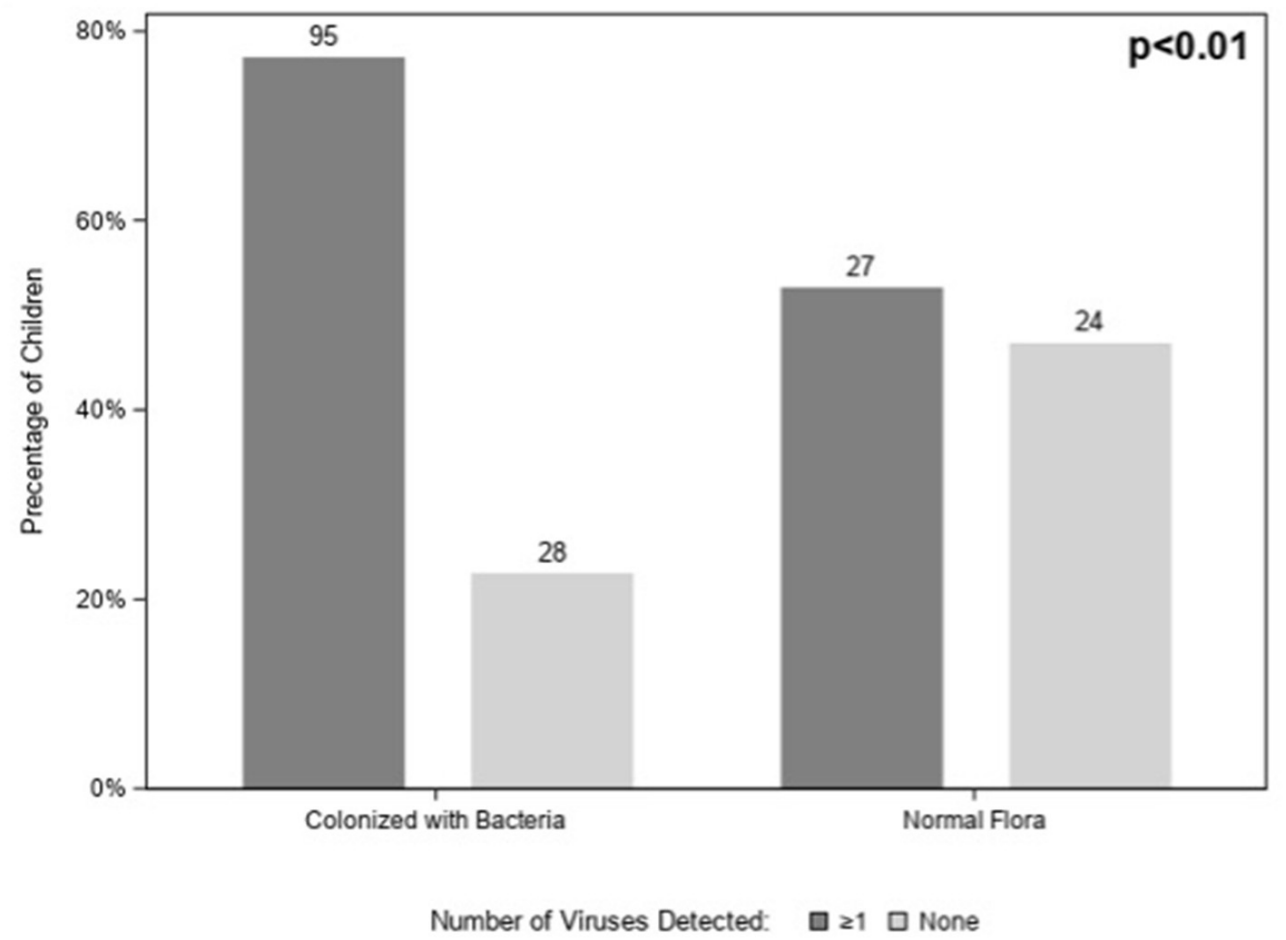
Number of viruses detected among children with acute sinusitis according to the presence of nasopharyngeal bacteria.

**Figure 3:**
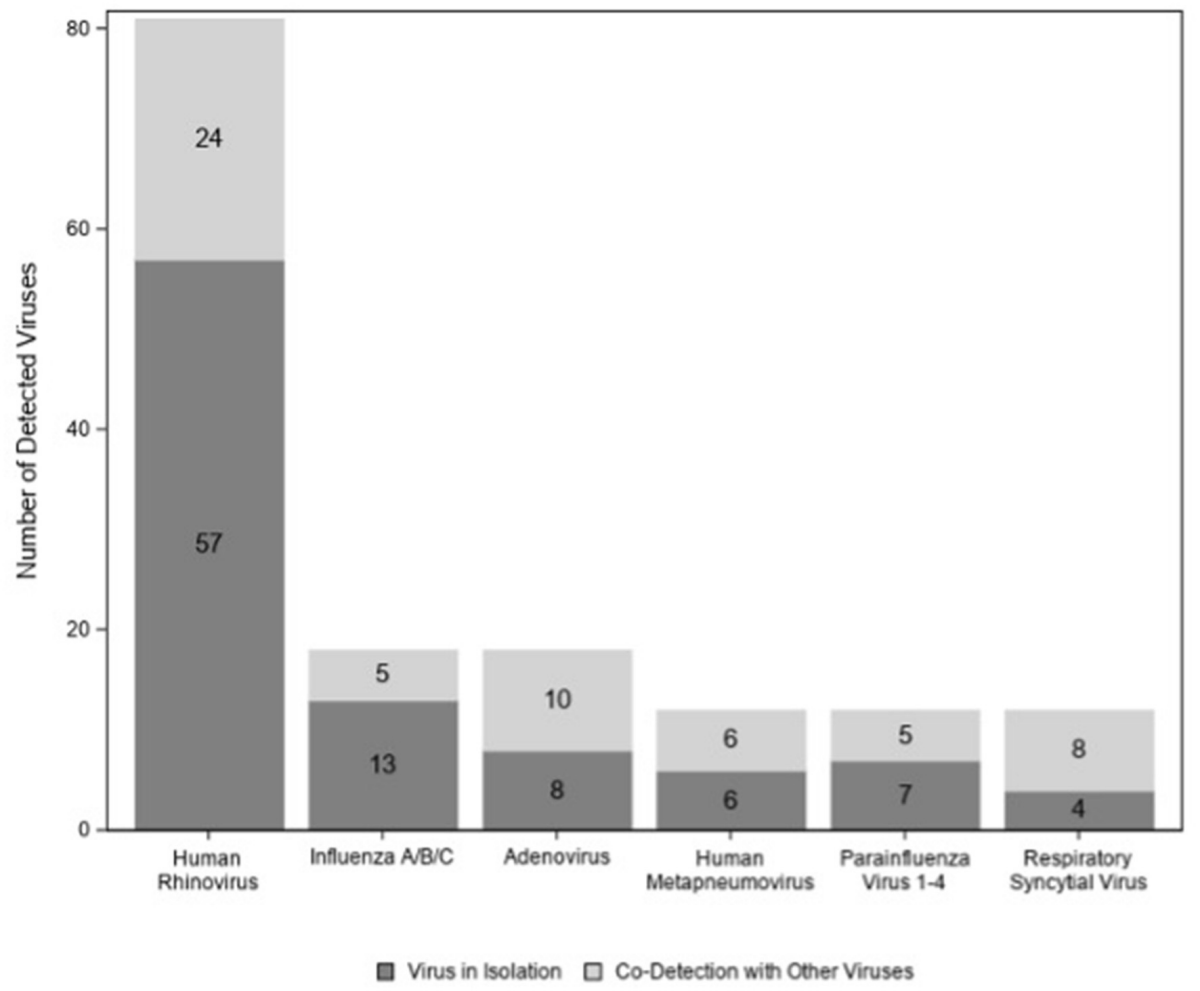
Individual viruses detected among children with acute sinusitis.

A total of 122/174 (70%) children tested positive for at least one virus. Viruses (*n* = 95; 77%) were more likely to be detected in children colonized with bacteria than in those with normal flora (*n* = 27; 53%) (*p* < *0.01*) (Figure 2); however, this association disappeared when we controlled for age (*p* = *0.06*) (data not shown). Among those 122 children with virus(es), HRV was the most commonly detected virus (*n* = 81) followed by influenza virus (*n* = 18) and adenovirus (*n* = 18) (Figure 3). There were no significant differences in the cycle threshold values between groups (data not shown).

### Cytokine Expression During Acute Sinusitis

We measured cytokine expression in 169 (97%) children, five children were excluded due to missing values on housekeeping protein (GAPDH). Cytokine mRNA levels were similar in children with normal flora and children colonized with bacteria (Table 2 and Supplementary Figure 1), and this remained the case in younger children (age ≤5 years old).

**Table 2:** Normalized cytokine expression in children with acute sinusitis according presence of bacterial pathogens in the nasopharynx.

**Common name**	**Gene name**	Colonized with bacteria^*^ (*n* = 118)	**Normal flora (*n* = 51)**	*P*-value^**^
			Median [IQR]	
**Pro-inflammatory**				
IL-6	*IL6*	3.9 [1.5, 18.0]	1.7 [0.9, 7.8]	0.12
IL-8	*CXCL8*	6.8 [2.3, 25.5]	2.2 [0.8, 19.4]	0.26
IL-17	*IL17A*	0.6 [0.3, 1.4]	0.9 [0.3, 1.6]	0.46
IL-22	*IL22*	11.2 [1.6, 44.1]	7.5 [0.3, 30.6]	0.40
IL-25	*IL25*	0.6 [0.1, 3.8]	1.5 [0.4, 13.4]	0.18
IL-26	*IL26*	0.5 [0.2, 1.0]	0.6 [0.3, 1.3]	0.20
IL-33	*IL33*	0.6 [0.2, 1.1]	0.8 [0.4, 1.4]	0.40
**Anti-inflammatory**				
IL-10	*IL10*	2.0 [0.9, 4.3]	1.4 [0.7, 4.5]	0.56
IL-13	*IL13*	0.7 [0.1, 6.3]	1.8 [0.2, 27.4]	0.12
sIL22-RA	*IL22RA2*	4.2 [1.6, 8.0]	2.8 [0.6, 7.6]	0.26
**Antiviral**				
IFN-α	*IFNA1*	2.1 [0.7, 8.5]	3.6 [0.9, 9.7]	0.47
IFN-β	*IFNB1*	3.5 [1.1, 11.7]	6.2 [1.7, 14.7]	0.40
IFN-γ	*IFNG*	0.9 [0.5, 2.2]	1.1 [0.4, 2.6]	0.58
IFN-λ	*IFNL1*	1.6 [0.3, 79.2]	3.3 [0.3, 234.0]	0.47

*IL, Interleukin; CXCL, chemokine (C-X-C motif) ligand; sIL22-RA, soluble receptor for IL-22; IFN, Interferon; IQR, interquartile range*.

* *Five children excluded due to missing values on housekeeping protein (GAPDH)*.

** *P-values were adjusted for multiple comparisons using the Benjamini-Hochberg method*.

### Independent Predictors of Bacterial Presence

Multivariate analysis found that colonized children were younger than children with normal flora (*p* < 0.01) (data not shown).

## Discussion

To our knowledge, this is the first study to characterize the presence of pathogens, viruses, and immune response in the nasopharynx at the time diagnosis of acute sinusitis in children. We found significant heterogeneity of bacteria isolated from the nasopharynx, with *M. catarrhalis* being the most common isolate. In addition, we identified viruses in a large proportion of children, with HRV being the predominant one. The initial insult for acute sinusitis is often a viral upper respiratory tract infection (9). We found that HRV was the most common virus detected during acute sinusitis, which is similar to other published data (27).

We found that viruses were more likely to be present in children colonized with bacterial pathogens (Figure 2). However, this difference was largely due to a difference in age in children with and without bacterial colonization; after controlling for age there was no significant association between viruses detected and presence of bacterial pathogens (*p* = 0.06).

Elevated levels of IL-1, IL-8, IL-6, and tumor necrosis factor-alpha (TNF-α) in nasal lavage fluid during acute viral upper respiratory tract infection have been reported (28, 29). Furthermore, a longitudinal study of 151 children with viral URI showed that elevated concentration of IL-6 correlated with fever and was significantly higher during influenza and adenovirus infection compared with enterovirus and HRV (30). IL-1 concentration was significantly higher in patients that later developed acute otitis media compared with subjects without acute otitis media (30). Unlike the above studies, nasal cytokine levels did not distinguish between children with and without bacterial colonization of the nasopharynx in this study. In addition, we found no difference on nasal cytokine levels between children using oral or topical antihistamine pre-enrollment (data not shown). Our findings describe an orchestrated immune response with overlapping proinflammatory and anti-inflammatory effects during acute sinusitis (Table 2). A better understanding of the host mucosal immune response could provide a basis for development of a novel diagnostic methodology.

Acute sinusitis is a clinical diagnosis based on stringent criteria with no ancillary test to aid the diagnosis (9). Differentiation between viral vs. bacterial infections can be challenging based on clinical presentation. To illustrate this, a recent longitudinal study found that 30% of patients with acute sinusitis had a new virus identified, with RSV the most common virus detected (31). Increasing availability of gene expression and transcriptional profiling could help with the diagnosis or management of acute sinusitis. A recent publication showed that host antiviral gene expression of CXCL10, CXCL 11, IFIT2, and OASL is associated with presence of respiratory viruses (32). Another showed the combination of tumor factor-related apoptosis-induced ligand (TRAIL), γ-induced protein-10 (IP-10), and C-reactive protein can differential bacterial infection from a viral process (33). Another group showed that IL-17, IL-4, IFN-γ, and INF-λ inducible protein-10 were associated with decreased risk of hospitalization during viral lower respiratory tract infection (29). Our cytokine expression results did not help differentiate between children with acute sinusitis who were colonized with pathogens and those who were not.

Our study has several limitations. Nasopharyngeal swabs are not routinely performed in clinical practice; however, we used this method as a research tool to evaluate its potential role in diagnosis. There was a statistically significant difference in the age between the children with and without bacterial colonization (Table 1). However, we found no differences in seasonality, clinical presentation, or disease severity between the groups. We excluded children with severe sinusitis presentation, but this is <5% of children with sinusitis (9). A strength of our study was inclusion of a large representative sample of children and development of a novel method of performing bacterial culture, viral detection, cytokine measurement using a single NP swab (11).

## Conclusion

This study yields insight into the host's response to viruses and bacteria in the nasopharynx. Further exploration of these interactions may elucidate the contributions of each to the pathophysiology of acute sinusitis.

## Data Availability Statement

The raw data supporting the conclusions of this article will be made available by the authors, without undue reservation.

## Ethics Statement

The studies involving human participants were reviewed and approved by University of Pittsburgh Institutional Review Board. Written informed consent to participate in this study was provided by the participants' legal guardian/next of kin.

## Author Contributions

NS, SL, and HL are responsible for drafting the initial manuscript. NS, SL, JM, and JW are responsible for study concept and design, acquisition of data analysis and interpretation of data, and formal analysis. HL and MJ are responsible for acquisition of data and analysis and interpretation of data. All authors revised the manuscript, approved the final manuscript as submitted, and agree to be accountable for all aspects of the work.

## Funding

This work was supported by NIH [U01AI118506]; www.clinicaltrials.gov, study identifier: [NCT02554383]. SL was supported by TL1 [TL1R001858]. JW was supported by the Henry L. Hillman Chair in Pediatric Immunology. This project was also supported by an Institutional Development Award (IDeA) from the National Institute of General Medical Sciences of the National Institutes of Health under grant number 5P20GM121341.

## Conflict of Interest

JW serves on the Scientific Advisory Board of Quidel and ID Connect, and on an Independent Data Monitoring Committee for GlaxoSmithKline, none related to the present work. The remaining authors declare that the research was conducted in the absence of any commercial or financial relationships that could be construed as a potential conflict of interest.

## Publisher's Note

All claims expressed in this article are solely those of the authors and do not necessarily represent those of their affiliated organizations, or those of the publisher, the editors and the reviewers. Any product that may be evaluated in this article, or claim that may be made by its manufacturer, is not guaranteed or endorsed by the publisher.
