# Supplementary figures and images for: Viral Coinfection and Nasal Cytokines in Children With Clinically Diagnosed Acute Sinusitis
Source: Front Pediatr. 2022 Jan 12;9:783665. doi: 10.3389/fped.2021.783665 (PMC8791629; doi:10.3389/fped.2021.783665)

**Supplementary Figure 1:**


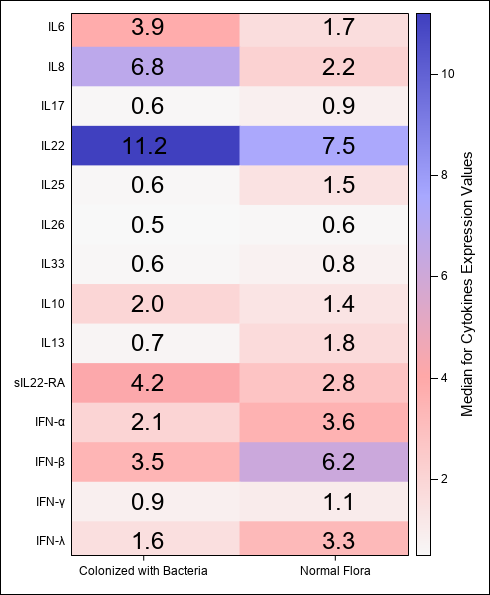

Supplement: Supplementary file 1 [file Table_1.DOCX]
